# Label-Free Aptasensor for Lysozyme Detection Using Electrochemical Impedance Spectroscopy

**DOI:** 10.3390/s18020354

**Published:** 2018-01-26

**Authors:** Dionisia Ortiz-Aguayo, Manel del Valle

**Affiliations:** Sensors and Biosensors Group, Department of Chemistry, Universitat Autònoma de Barcelona, Bellaterra 08193, Spain; Dionisia.ortiz@uab.cat

**Keywords:** aptamer, biosensor, electrochemical impedance spectroscopy, electrochemical grafting

## Abstract

This research develops a label-free aptamer biosensor (aptasensor) based on graphite-epoxy composite electrodes (GECs) for the detection of lysozyme protein using Electrochemical Impedance Spectroscopy (EIS) technique. The chosen immobilization technique was based on covalent bonding using carbodiimide chemistry; for this purpose, carboxylic moieties were first generated on the graphite by electrochemical grafting. The detection was performed using [Fe(CN)_6_]^3−^/[Fe(CN)_6_]^4−^ as redox probe. After recording the frequency response, values were fitted to its electric model using the principle of equivalent circuits. The aptasensor showed a linear response up to 5 µM for lysozyme and a limit of detection of 1.67 µM. The sensitivity of the established method was 0.090 µM^−1^ in relative charge transfer resistance values. The interference response by main proteins, such as bovine serum albumin and cytochrome c, has been also characterized. To finally verify the performance of the developed aptasensor, it was applied to wine analysis.

## 1. Introduction

Nowadays, there is an undoubted need of monitoring and controlling different parameters in fields such as food industry, forensic, environmental monitoring, drug development or clinical diagnoses. Conventional analytical methods may provide high sensitivity and selectivity but they can present problems such as time-delays, high cost and have requirements of trained personal. Thus, it is very important to find reliable analytical devices capable of performing accurate and fast analyses. One proposal to overcome these problems is the development of biosensors [1]. Biosensors can be classified, depending on the technique employed for the transduction, as optical, calorimetric, piezoelectric and electrochemical or, by the type of biorecognition element, as enzyme, nucleic acid, aptamer, cells and biomimetic systems. In this work, the developed biosensors are aptasensors and the type of transduction for biosensing is electrochemical. Aptasensors are biosensors that use aptamer [2] as the biorecognition element. Aptamers (from the Latin *aptus*–fit, and Greek *meros*–part) are artificial single strands of DNA or RNA with a length in the range of 10–100 nucleotides, which are selected in vitro and have the ability, through specific folding conformations, to bind proteins, ions, whole cells, drugs and low molecular weight ligands, ranging from picomolar (pM) to nanomolar (nM) level. They recognize their target with high affinity and specificity [3], often matching or even exceeding those of antibodies [4]. Their affinity constants reach the order of 3 × 10^7^ M^−1^, which makes them promising recognition agents. Due to all these properties, aptamers can be used in a wide range of applications, such as therapeutics and diagnostics [5], molecular switches, drug development, affinity chromatography and biosensors [6,7]. The in vitro process [8] to obtain a certain specific aptamer is called SELEX (*Systematic Evolution of Ligands by Exponential enrichments*). Therefore, this study describes the construction of an electrochemical aptamer-based sensor (aptasensor) for lysozyme (Lys) detection using Electrochemical Impedance Spectroscopy (EIS) technique. EIS [9] has been used in many fields of electrochemistry, electrode kinetics, double-layer studies, batteries, corrosion, solid-state electrochemistry and bioelectrochemistry [10]. In addition, it can be considered as a characterization technique, which provides electric information in the frequency domain. With this technique, a process that occurring in an electrochemical cell can be modelled using a combination of electrical circuits that give the same AC current response provided by the electrochemical system [11]. By the use of equivalent circuits, the experimental spectra can be fitted with the theoretical curve corresponding to the selected circuit model, thus obtaining the values of electrical parameters (resistance, capacitance, etc.) which are directly correlated to specific electrochemical phenomena occurring in the system under study [10]. Its wide ability for the characterization of electrode-electrolyte interfaces and its high sensitivity for probing the interfacial properties of a modified electrode surface have made EIS a good alternative for biosensing during the last years. Besides, an important feature presented by the EIS technique is that it does not require any labelled species for the transduction. Thus, it can be used for designing label-free protocols avoiding more expensive and time-consuming assays.

The analyte studied in the present work was lysozyme (Lys). Lysozyme is a mucopolysaccharide alkaline enzyme that is capable of destroying bacterial cellular membranes by catalyzing the breakage of the β-1,4 bond found in peptidoglycan residues of cell walls of Gram-positive bacteria [12]. This protein has many points of interest, because it is considered as a good model to study enzyme catalysis, protein structure and interactions or amyloid-fibrillation formation. In addition, Lys from hen egg has become also a model protein for the pharmaceutical industry when it comes to the development of new drug delivery systems or the design of innovative treatment strategies. Furthermore, Lys is involved in different fields such as wine-making and food industry including cheese and beer production. In the case of wine industry, this protein replaces sulfites and it is added at doses of 250–500 mg·L^−1^ to inhibit malolactic fermentation and to stabilize the wine afterwards. Residual levels of 0.06–327 mg·L^−1^ were found in lysozyme-treated wines [13]. According with the International Organization of Vine and Wine (OIV) the maximum permissible dose of Lys in wine-making is 500 mg·L^−1^ (~35 μM) [14], thus it is considered as regulated substance. Despite the above, Lys has many drawbacks associated to allergic reactions in susceptible individuals and elevated human Lys level in serum and urine may cause kidney problems [15] and leukemia [16]. At last, there are different analytical methods [17] for its detection such as chromatography and ELISA among others, but they are expensive and need complex procedures. For this reason, there is a need to develop rapid, cheap and effective devices for Lys detection. All of these requirements are supplied by label-free aptasensors [18].

As mentioned previously, we have designed in this work a disposable and label-free aptasensor for the detection of Lys protein. To achieve this feature, different optimizations were performed, a calibration curve of the sensor and its behavior were also assessed in a complex matrix such as wine. The specificity and the regeneration of the sensor were also evaluated. The transduction principle used is based on the change of electron-transfer resistance in the presence of a redox probe, which can be measured by EIS. The aptamer was immobilized by covalent binding using carbodiimide chemistry [19,20]. The aptasensor showed a high sensitivity, good specificity and they have a low production cost and rapid response.

## 2. Materials and Methods

### 2.1. Chemicals and Reagents

Potassium ferricyanide K_3_[Fe(CN)_6_], potassium ferrocyanide K_4_[Fe(CN)_6_], sodium chloride (NaCl), potassium chloride (KCl), disodium phosphate (Na_2_HPO_4_) were obtained from Merck (Darmstadt, Germany). N-hydroxysuccinimide (NHS), N-(3-dimethyl aminopropyl)-N′-ethylcabodiimide hydrochloride (EDC), poly (ethylene glycol) 1000 (PEG) were obtained from Fluka (Buchs, Switzerland). Monopotassium phosphate (KH_2_PO_4_), sodium nitrite (NaNO_2_), MES monohydrate, 4-aminobenzoic acid (ABA) and magnesium chloride (MgCl_2_) were obtained from Sigma Aldrich (St. Louis, MO, USA). Graphite-epoxy composite electrodes (GEC) were prepared using 50 μm particle size graphite powder (Merck, Darmstadt, Germany), Resineco epoxy resin and its corresponding hardener. Biomers (Ulm, Germany) was the source of the aptamer used in this study. It has a modification in 5′ extreme with aminolink C6. The sequence is 5′-ATC TAC GAA TTC ATC AGG GCT AAA GAG TGC AGA GTT ACT TAG-3′ for proper immobilization using an amide linkage as described in the literature [18]. Lysozyme (Lys), bovine serum albumin (BSA) and cytochrome c from bovine heart (Cyt c) were purchased from Sigma Aldrich (St. Louis, MO, USA). Commercial wine samples used are from Don Simon. All solutions were made up using sterilized Milli-Q water (Millipore, Billerica, MA, USA). The buffer solutions employed were PBS (187 mM NaCl, 2.7 mM KCl. 8.1 mM Na_2_HPO_4_·H_2_O and 1.76 mM KH_2_PO_4_, pH 7.0), BB (1 mM MgCl_2_, 2.7 mM KCl, 140 mM NaCl, 0.1 mM Na_2_HPO_4_ and 1.8 mM KH_2_PO_4_, pH 7.0) and MES (100 mM 2-(N-morpholino)ethanesulfonic acid and 0.09% NaCl, pH 7.0). Aptamer stock solutions were first diluted with deionized water, separated in fractions of 10 μM and stored at a temperature of −20 °C. When required, a single fraction was defrosted and used.

### 2.2. Equipment

Alternating current (AC) impedance measurements were performed with an Autolab PGStat 20 (Metrohm Autolab B.V, Utrecht, The Netherlands). FRA software was used for the acquisition of the data and the control of the experiments. Finally, ZView (Scribner Associates Incorporated, Southern Pines, NC, USA) software was used for data processing. Cyclic Voltammetry measurements were performed with an Autolab PGStat 20 (Metrohm Autolab B.V, Utrecht, The Netherlands). GPES software was used for the acquisition of the data. A three-electrode cell was used to perform the impedance and cyclic voltammetry measurements: a platinum-ring auxiliary electrode (Crison 4.75, Barcelona, Spain), an Ag/AgCl reference electrode and GEC as working electrode. Furthermore, other equipment such as Eppendorf Thermomixer C to control temperature incubations were used for the experiment, pH-meter GLP22 (Crison 5224, Barcelona, Spain), Vortex shaker MS3 basic (IKA, Staufen, Germany) and Sterilizer CertoClav-Tisch-Autoclav 12L GS (Schaffhausen, Switzerland).

### 2.3. Preparation of Working Electrodes

The construction of the electrodes was carried out using PVC tube body (6 mm i.d.) and a small copper disk was soldered at the end of an electrical connector. The next step of the procedure is to prepare the graphite-epoxy composite mixture. This mixture was prepared by hand mixing of the epoxy resin, the hardener and the graphite powder [21,22]. The resulting paste was homogenized by mixing for 1 h and then it was cured at 40 °C during 2 d. Before each use, the electrode surface was moistened with MilliQ water and then thoroughly smoothed with abrasive sandpaper and finally with alumina paper (polishing strips 301044-001, Thermo Scientific Orion, Waltham, MA, USA) in order to obtain a reproducible electrochemical surface.

### 2.4. Experimental Procedure

**Figure 1:**
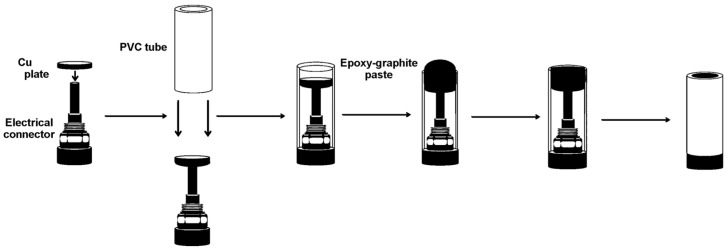
Construction of working electrodes based on graphite-epoxy composite.

**Figure 2:**
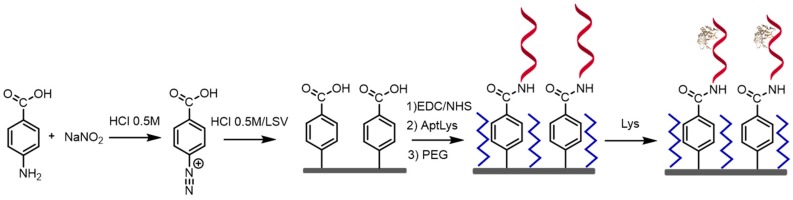
Steps for the experimental protocol for the label-free aptasensor for Lys detection based on covalent bond immobilization technique via electrochemical grafting.

The experimental procedure for biosensing consists of immobilization of the aptamer onto the transducer surface using carbodiimide chemistry via electrochemical grafting, followed by the labeless recognition of the Lys protein by its aptamer via incubation at room temperature. The steps of the working electrode construction and biosensing are described in more detail below (Figure 1 and Figure 2).

#### 2.4.1. Aptamer Preconditioning

Before aptamer immobilization, it is very important to make a pretreatment of the aptamer. This aptamer preconditioning is carried out in order to promote its loose conformation. Thus, the aptamer solutions were heated at 80–90 °C for 3 min. Then, the solution was dipped in a bath of cold water to fix the loose conformation.

#### 2.4.2. Aptasensor Immobilization onto the Electrode Surface

After the preconditioning of the aptamer, the aptamer is immobilized through a covalent bond between the aptamer and the electrode surface. This is done by electrochemical grafting [23], being its goal to obtain stable carboxyl-derivatized conductive surfaces for the immobilization of aminated biomolecules. To achieve this, 4-aminobenzoic acid (ABA) and sodium nitrite are used to form a diazonium salt, which is immobilized with electrochemical control on the electrode surface. Afterwards, the amino terminated aptamer is immobilized through formation of an amide bond helped with the carbodiimide chemistry. In detail, the GEC was first subjected to electrochemical pretreatment by 10 cyclic potential scans between 1.0 and −1.5 V at scan rate of 0.2 V/s in 0.5 M H_2_SO_4_ and 0.1 M KCl in order to activate the surface. Then, the diazotation reaction took place adding 40 μL of 1 M NaNO_2_ and 20 mL of 2 mM ABA prepared in 0.5 M HCl. The mixture was left to react for 5 min at low temperature [24]. After that, 10 mL of the mixture and 10 mL of 0.5 M HCl were deposited onto the electrode surface and the electrochemical modification was performed by 10 linear sweep voltammetry scans from 0.6 to −0.8 V. After this modification, the electrode was rinsed three times with distilled water. After that, a solution using EDC reagents (100 mM EDC and 25 mM NHS in 100 mM MES buffer) is prepared in order to activate the carboxylic groups. Then, 130 μL of EDC reagents are added in an Eppendorf jointly with 30 μL of aptamer 1 μM. The incubation is performed for 12 h. The next day, the electrodes were washed twice with BB buffer during 5 min to remove unbound Apt.

#### 2.4.3. Blocking Step

After that, a blocking step is performed using polyethylene glycol as blocking agent; the assay to detect Lys consists of a simple incubation step with the sample and non-specific adsorption of unwanted species needs to be avoided. For the latter, the electrode surface is blocked by submerging in 160 μL of 50 mM polyethylene glycol (PEG) solution for 20 min at 25 °C. Next, two 10 min washing steps using BB solution were followed.

#### 2.4.4. Label Free Detection of Lysozyme

The last step of the procedure consists of the recognition between the aptamer and the protein. For the detection of the target protein, the Apt modified electrodes were incubated for 1 h with the desired concentration of Lys and finally the aptasensor was washed twice for 10 min with BB solution.

#### 2.4.5. Regeneration of Aptasensor

To carry out the regeneration of the aptasensor the following protocol was used. The first step is to dissociate the AptLys-Lys complex. To achieve this objective, the used aptasensor is maintained in stirring conditions in saline media (NaCl) and increasing the temperature until 42 °C.

### 2.5. Impedimetric Measurements and Data Processing

**Figure 3:**
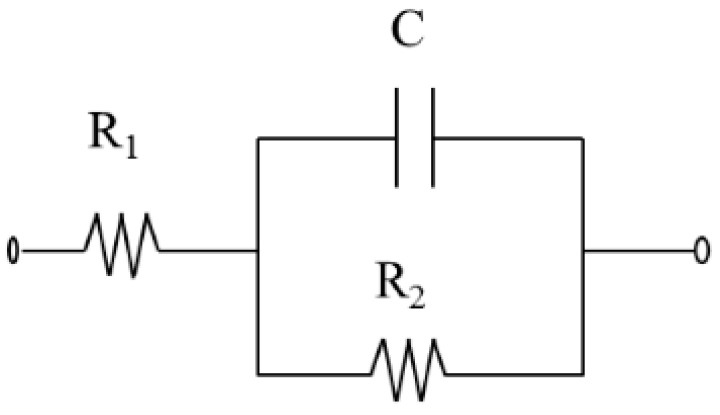
Randles equivalent circuit proposed to best fit.

All the experiments were performed in PBS buffer containing 0.01 M K_3_[Fe(CN)_6_]/K_4_[Fe(CN)_6_] (1:1) mixture as redox marker. The applied potential was +0.17 V (vs. Ag/AgCl reference electrode) and the range of frequency of 50 KHz–0.05 Hz, an AC amplitude of 10 mV and a sampling rate of 10 points per decade above 66 Hz and 5 points per decade at the lower range. The most common graphical representation of impedimetric data is the “Nyquist Diagram”, in which the imaginary part of the impedance (−*Z_i_*), is plotted versus the real part *Z_r_*. The equivalent Randles circuit [25] proposed to fit the experimental data is shown in Figure 3 where *R*_1_ is the resistance of the solution, *R*_2_ is the charge transfer resistance associated to the redox reaction, that established between the solution and the electrode surface, and *C* is the capacitance. In our case, a constant phase element (CPE) is used instead of a capacitor. CPE is required to optimize the fit to the experimental data, and this is due to the nonideal nature of the electrode surface. In this treatment, the diffusion component was discarded, as it is non-relevant for the purpose of the study. In our case, we are interested in two parameters: the charge transfer resistance (*R*_2_) and the chi-square (χ^2^). Both are obtained by ZView software. The first one evaluates the modification of the electrode surface, while the chi-square evaluates the goodness of fitting. As in all cases, the calculated values of chi-square for each experiment is <0.2, much lower than the tabulated value for 60 degrees of freedom (67.505 at 95% confidence level), that verifies that the adjustments were done correctly. With the aim of comparing the results obtained from the different electrodes used, a normalization is performed. This normalization is called Δ_ratio_ [6,22], whose advantage is the improvement of the reproducibility of the measurements. It was calculated as follows:

Δ_ratio_ = Δ_s_/Δ_p_ (1)

Δ_s_ = *R*_*ct*(*Apt-Prot*)_ − *R*_*ct*(*electrode-bare*)_ (2)

Δ_p_ = *R*_*ct*(*Apt*)_ − *R*_*ct*(*electrode-bare*)_ (3)

In this case, *R*_*ct*(*Apt-Prot*)_ is the electron transfer resistance measured after incubating with the protein desired (Lys), *R*_*ct*(*Apt*)_ is the electron transfer resistance after immobilization and PEG blocking and *R*_*ct*(*electrode-bare*)_ is the electron transfer resistance of the bare electrode and buffer. In future experiments the measurements were expressed as Δ_ratio_.

## 3. Results and Discussions

### 3.1. Incubation Time of Aptamer

**Figure 4:**
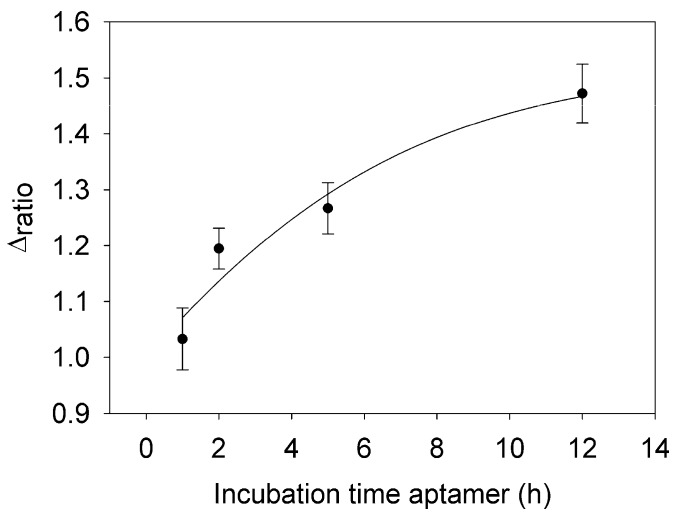
Optimization of the incubation time of aptamer using electrochemical grafting as immobilization technique.

This section was developed in order to optimize the immobilization time of aptamer employed. For this, four different incubation times such as 1.0 h, 2.0 h, 5 h and 12 h were evaluated. The concentrations of the aptamer and the blocking agent used were 1 μM and 40 mM, respectively. As it shows Figure 4, the minimum incubation time of the aptamer that can be used is 2 h, due to the Δ_ratio_ begins to increase. Less than 2 h is not a good immobilization time of the aptamer onto the surface of the electrode, as the Δ_ratio_ values are close to 1. For that reason and for practical use, the best option considered was 12 h. In this time, high Δ_ratio_ values were obtained and also with proper reproducibility.

### 3.2. Optimization of Aptamer and PEG Blocking Agent Concentrations

**Figure 5:**
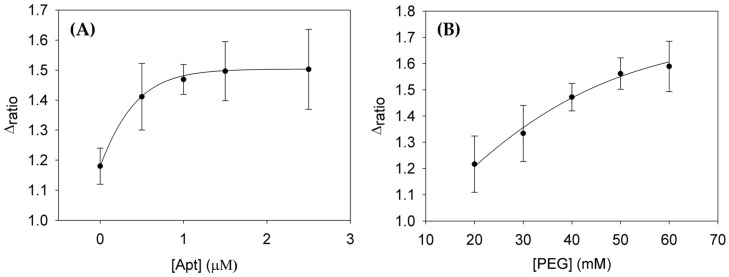
(**A**) Optimization of the concentration of AptLys; (**B**) Optimization of the concentration of the blocking agent, PEG. Uncertainly values corresponding to replicated experiments (*n* = 5).

First, the concentration of the aptamer and the blocking agent must be optimized by building its interaction curves. To perform these experiments 0.05, 0.5, 1 and 2.5 μM concentrations of aptamer were tested. The concentration of the Lys and PEG employed were 2.5 μM and 40 mM, respectively. In Figure 5A, the interaction curve of AptLys immobilized onto the electrode surface is represented. In this plot, it can be observed that the values of Δ_ratio_ increase until a fixed value is reached. This phenomenon is produced because it corresponds to a Langmuir isotherm. From a concentration above 1 μM the saturation of the aptamer on the electrode surface is produced, therefore the optimum concentration established to perform the calibration curve of Lys was 1 μM. In order to avoid nonspecific adsorption the concentration of blocking agent needs to be optimized as well. In this case, the blocking agent selected was PEG and the concentrations tested were 20, 30, 40, 50 and 60 mM. As it shown in Figure 5B, the behavior is similar to the previous case. The values of Δ_ratio_ increase until the concentration tested is 60 mM; this fact shows that PEG followed an adsorption isotherm. Hence, the optimum concentration of PEG was 50 mM.

### 3.3. Analytical Performance of the Aptasensor for Lysozyme Detection

**Figure 6:**
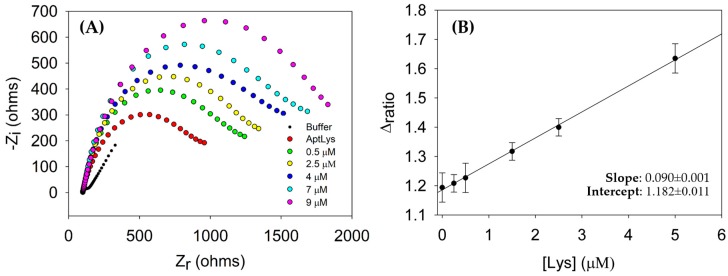
(**A**) Nyquist Plot for a biosensing protocol with increasing Lys concentrations: 0.5, 2.5, 4, 7 and 9 μM; (**B**) Calibration curve vs. Lys concentrations: 0, 0.25, 0.5, 1.5, 2.5 and 5 μM. Uncertainty values corresponding to replicated experiments (*n* = 5). All the measurements were performed in PBS solution in the presence of the redox probe.

The analytical performance of the aptasensor was evaluated with different concentrations of Lys. For this, the concentration of Lys employed were 0.25, 0.5, 1.5, 2.5 and 5 μM. The concentrations of aptamer and PEG employed were 1 μM and 50 mM, respectively. The aptamer strand recognizes the protein through a three-dimensional folding following host-guest principles. The results obtained in this experiment are shown in Figure 6A. In this representation, it can be seen that the charge-transfer resistance *R_ct_* increased in each step of the procedure. This is due to the effect on the kinetics of the electron transfer reaction by the redox probe, which is delayed at the interface of the electrode, mainly caused by steric hindrance and electrostatic repulsion presented by the complex aptamer-protein formed [26]. This is an additional advantage of using EIS as the transduction principle: each step in the biosensing protocol can be assessed, thus confirming the procedure is correctly performed. On the other hand, when the concentration of Lys increases the *R_ct_* also increases. To evaluate the detection limit (LOD), sensitivity and the lineal response range, a calibration curve was built. In this calibration curve (Figure 6B) the analytical signal (Δ_ratio_) is plotted versus the concentration of Lys. In a first instance, it is deduced that there is a correct recognition of the protein by the aptamer. This fact is observed when the concentration of Lys increases because, *R*_2_, the interfacial electron transfer resistance between the electrode surface and solution increases as well. The linear relationship (*R* = 0.999) between the Δ_ratio_ and the concentration of Lys is according to the equation: Δ_ratio_ = 0.090 [Lys] + 1.182. All the measurements were performed with *n* = 5 replicates. The detection limit, 1.67 μM, was estimated as 3.3 times the standard deviation (*n* = 5) of the lowest stock concentration (0.25 μM) and the sensitivity (0.090 μM^−1^) is the slope of the calibration curve, all these in label-free conditions. In addition, it was obtained a calibration curve with a linear range from 1.67 μM to 5 μM of Lys. However, Figure 6B shows perceptible signal below the LOD, because it was calculated using the standard deviation of the lowest stock concentration, what increase noticeable the LOD.

### 3.4. Regeneration of the Aptasensor

**Figure 7:**
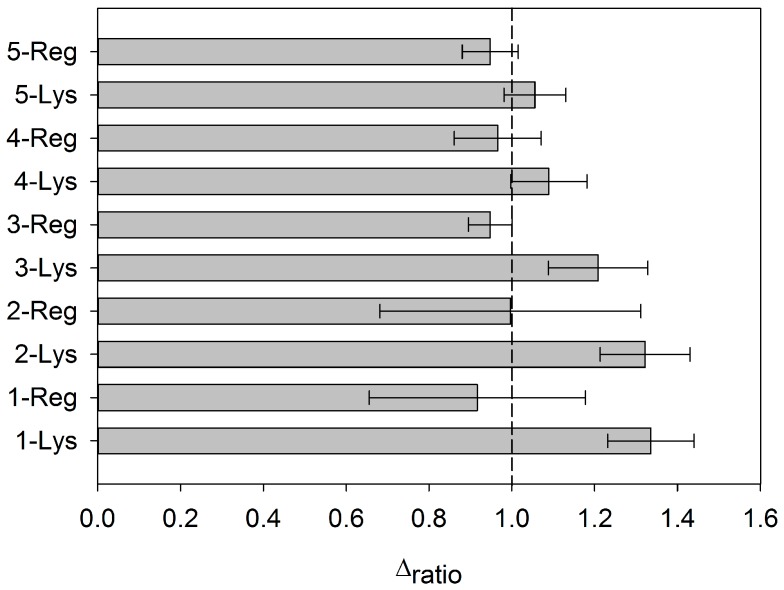
Δ_ratio_ values for five consecutive cycles of regeneration. The concentration of Lys employed is 2 μM. The deviation is calculated for *n* = 5 replicates.

It was possible to regenerate the aptasensor by dissociating the AptLys-Lys complex. This was achieved by stirring the aptasensor in saline media (NaCl) and increasing the temperature [27]. At this point, the complex is dissociated and the resistance decreased to the baseline value of the correspondent AptLys. In the study, five sensing cycles were evaluated with a blank measure in between each one. As it shows in Figure 7, the aptasensor could regenerate twice at most. This fact is observed with the first and second regeneration. From the third regeneration, the detection of Lys is lower, probably due to the loss of aptamer from the electrode surface. For this reason, this regeneration is not carried out in the performed assays, which were done on each cycle with brand new immobilization of the aptamer on each cycle. To recover electrode use for future experiments, the followed option was to perform a surface polishing and new immobilization.

### 3.5. Specificity of the Aptasensor

**Figure 8:**
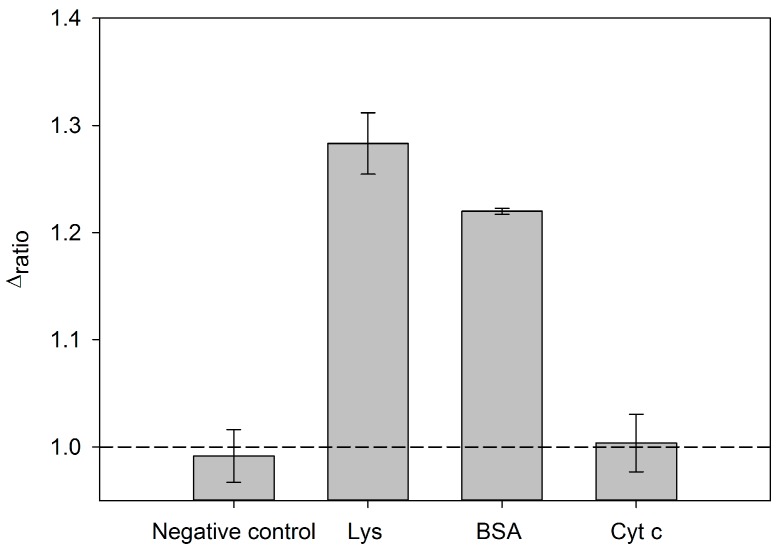
Specificity of AptLys to Lys, BSA, Cyt c at 0.5 μM. Errors bars are obtained based on five independent measurements.

An efficient electrochemical aptasensor has to display good specificity. The influences of interferences can be estimated from the value of the delta ratio of assays in their presence. In order to verify that the aptasensor developed is specific for the detection of Lys different experiments were performed. The proteins analyzed were Lys, Cyt c and BSA, essentially because they were common proteins and available in the laboratory. The procedure was carried in the same manner as above but changing the protein in the last step of the protocol. The concentration of the protein used in these selectivity assays was 0.5 μM. The concentrations of aptamer and PEG used were those optimized in the previous sections. The results are shown in Figure 8. As it is observed there, Cyt c is not detected by the aptasensor. Alternatively, in the case of BSA, the interfering effect is more significant. It is important to highlight that the interference given by proteins depends of the blocking agent used, as it is originated on the non-specific adsorption of the former. In our protocol, the employed blocking agent was PEG, normally considered a weak blocking agent. In the literature, many researchers reported the use of stronger blocking agents such as casein or BSA. One possible solution to improve the assay and overcome the BSA interference would be to use a stronger blocking agent such as the same BSA or casein, which would occupy the adsorption points. This alternative would be necessary only if a very complex sample matrix were used. To conclude, a negative control was also evaluated with the aim of verifying that the protocol operates properly. This negative control was made with deionized water and it demonstrated that the procedure provided consistent results.

### 3.6. Application of the Aptasensors in Spiked Wine Samples

To check the feasibility of the developed aptasensor, it was employed to evaluate Lys in real samples such as wine. For this, wine aliquots were spiked with Lys 1 μM using the developed aptasensors. The obtained recovery rate is illustrated in Table 1. The developed aptasensors exhibit good recovery of 77% indicating the suitability for Lys detection in wine samples. The relative low recovery was obtained probably due to some matrix interference. In spite of, the aptasensors still meet the criteria and merits of biosensors in terms of recovery.

## 4. Conclusions

This research reports a simple label-free aptasensor for the detection of lysozyme. The electrochemical grafting using carbodiimide chemistry has been a good choice for the immobilization due to its ease of realization, and for the possibility of electrical addressing. With the use of EIS technique we can evaluate the recognition event, and check the rest of the processes modifying the electron transfer kinetics of the redox probe at the electrode interface. In addition, the described aptasensor showed a reasonable range of response (from 1.67 μM to 5 μM) with a lower detection limit (LOD) of 1.67 µM. The calculated LOD is lower than the maximum amount allowed to be added in wine as prescribed by the International Organization of Vine and Wine (OIV), allowing the determination in this field. The interference produced by BSA showed distorted signal that probably may be solved using stronger blocking agent. Unfortunately, thermal wet regeneration of the biosensor was not effective for more than twice, impeding its repeated use. Finally, the aptasensor developed in this research could be applied for the detection of lysozyme in a complex matrix such as wine, providing satisfactory recovery yields of about 77%, showing the great potential of the proposed methodology for detecting Lys in other food matrices.

## Figures and Tables

**Table 1:** Recoveries of Lys in wine samples of aptasensors in real matrix such as wine.

Aptasensor	Spike Lys (µM)	Δ_ratio_	Found Lys (µM)	Recovery (%)
Apt Lys	0	1.19	0	-
	1	1.25	0.77	77
